# *trans*-3-(3,4-Dimeth­oxy­phen­yl)-2-(4-nitro­phen­yl)prop-2-ene­nitrile

**DOI:** 10.1107/S1600536810023196

**Published:** 2010-06-23

**Authors:** Abdullah M. Asiri, Salman A. Khan, Kong Wai Tan, Seik Weng Ng

**Affiliations:** a Chemistry Department, Faculty of Science, King Abdul Aziz University, PO Box 80203, Jeddah 21589, Saudi Arabia; b Department of Chemistry, University of Malaya, 50603 Kuala Lumpur, Malaysia

## Abstract

The asymmetric unit of the title compound, C_17_H_14_N_2_O_4_, contains two independent mol­ecules in which the benzene rings are in a *trans* arrangement with respect to the C=C double bond and the rings are inclined by 4.3 (1) and 22.1 (1)° with respect to each other.

## Related literature

For the crystal structure of α-((4-meth­oxy­phen­yl)methyl­ene)-4-nitro­benzene­acetonitrile, see: Vrcelj *et al.* (2002 ▶). For background literature on this class of pigments, see: Asiri (1999 ▶).
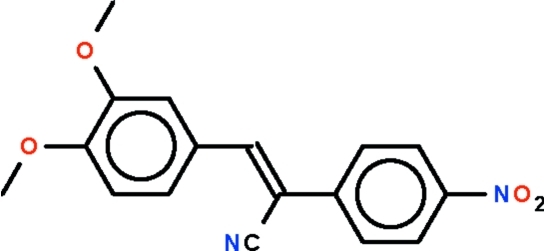


## Experimental

### 

#### Crystal data

C_17_H_14_N_2_O_4_
*M* *_r_* = 310.30
Triclinic, 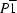

*a* = 10.2211 (8) Å
*b* = 11.9460 (9) Å
*c* = 12.2764 (10) Å
α = 91.094 (1)°
β = 99.542 (1)°
γ = 100.156 (1)°
*V* = 1453.3 (2) Å^3^
*Z* = 4
Mo *K*α radiation
μ = 0.10 mm^−1^
*T* = 100 K
0.40 × 0.20 × 0.10 mm

#### Data collection

Bruker SMART APEX diffractometer
13853 measured reflections
6628 independent reflections
4851 reflections with *I* > 2σ(*I*)
*R*_int_ = 0.034

#### Refinement

*R*[*F* ^2^ > 2σ(*F* ^2^)] = 0.047
*wR*(*F* ^2^) = 0.133
*S* = 1.02
6628 reflections
415 parameters
H-atom parameters constrained
Δρ_max_ = 0.40 e Å^−3^
Δρ_min_ = −0.24 e Å^−3^

### 

Data collection: *APEX2* (Bruker, 2009 ▶); cell refinement: *SAINT* (Bruker, 2009 ▶); data reduction: *SAINT*; program(s) used to solve structure: *SHELXS97* (Sheldrick, 2008 ▶); program(s) used to refine structure: *SHELXL97* (Sheldrick, 2008 ▶); molecular graphics: *X-SEED* (Barbour, 2001 ▶); software used to prepare material for publication: *publCIF* (Westrip, 2010 ▶).

## Supplementary Material

Crystal structure: contains datablocks global, I. DOI: 10.1107/S1600536810023196/lh5073sup1.cif

Structure factors: contains datablocks I. DOI: 10.1107/S1600536810023196/lh5073Isup2.hkl

Additional supplementary materials:  crystallographic information; 3D view; checkCIF report

## Figures and Tables

**Fig. 1.**
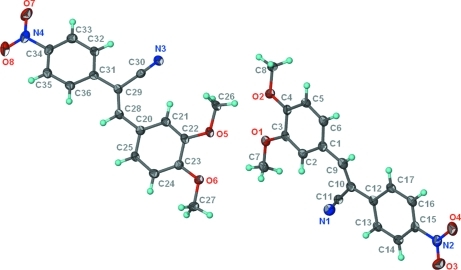
Thermal ellipsoid plot (Barbour, 2001) of C17H14N2O4 at the 70% probability level; hydrogen atoms are drawn as spheres of arbitrary radius.

## supplementary crystallographic information

### Comment

Organic photochromic compounds having donor and acceptor parts that are conjugated are potential optical materials. Such compounds, as exemplified by the title compound, are synthesized from carbonyl compounds having an active methylene group by using the Knovenagel condensation (Asiri, 1999). The title compound (Fig. 1) features a double-bond with two aromatic substituents in *trans*-positions.

α-((4-Methoxyphenyl)methylene)-4-nitrobenzeneacetonitrile, a stilbene derivative, exists in a *cis* and a *trans* form; interestingly, the *trans* form crystallizes in three modifications (Vrcelj *et al.*, 2002).

### Experimental

3,4-Dimethoxybenzaldehyde (0.41 g, 2.5 mmol) and 4-nitrobenzyl cyanide (0.40 g, 2.5 mmol) were heated in ethanol (15 ml) for 3 h; several drops of pyridine were added. The reaction was monitored by TLC. The solution was cooled and the residue rerystallized from a methanol-chloroform (1/1) mixture.

### Refinement

H-atoms were placed in calculated positions [C–H 0.95 to 0.98 Å, *U*(H) 1.2 to 1.5*U*_eq_(C)] and were included in the refinement in the riding model approximation.

### Crystal data

**Table:**

C_17_H_14_N_2_O_4_	*Z* = 4
*M**_r_* = 310.30	*F*(000) = 648
Triclinic, *P*1	*D*_x_ = 1.418 Mg m^−^^3^
Hall symbol: -P 1	Mo *K*α radiation, λ = 0.71073 Å
*a* = 10.2211 (8) Å	Cell parameters from 4130 reflections
*b* = 11.9460 (9) Å	θ = 2.4–28.1°
*c* = 12.2764 (10) Å	µ = 0.10 mm^−^^1^
α = 91.094 (1)°	*T* = 100 K
β = 99.542 (1)°	Prism, orange
γ = 100.156 (1)°	0.40 × 0.20 × 0.10 mm
*V* = 1453.3 (2) Å^3^	

### Data collection

**Table:**

Bruker SMART APEX diffractometer	4851 reflections with *I* > 2σ(*I*)
Radiation source: fine-focus sealed tube	*R*_int_ = 0.034
graphite	θ_max_ = 27.5°, θ_min_ = 1.7°
ω scans	*h* = −13→13
13853 measured reflections	*k* = −13→15
6628 independent reflections	*l* = −15→15

### Refinement

**Table:**

Refinement on *F*^2^	Primary atom site location: structure-invariant direct methods
Least-squares matrix: full	Secondary atom site location: difference Fourier map
*R*[*F*^2^ > 2σ(*F*^2^)] = 0.047	Hydrogen site location: inferred from neighbouring sites
*wR*(*F*^2^) = 0.133	H-atom parameters constrained
*S* = 1.02	*w* = 1/[σ^2^(*F*_o_^2^) + (0.0681*P*)^2^ + 0.1844*P*] where *P* = (*F*_o_^2^ + 2*F*_c_^2^)/3
6628 reflections	(Δ/σ)_max_ = 0.001
415 parameters	Δρ_max_ = 0.40 e Å^−^^3^
0 restraints	Δρ_min_ = −0.24 e Å^−^^3^

### Special details

**Table:**

Geometry. All e.s.d.'s (except the e.s.d. in the dihedral angle between two l.s. planes) are estimated using the full covariance matrix. The cell e.s.d.'s are taken into account individually in the estimation of e.s.d.'s in distances, angles and torsion angles; correlations between e.s.d.'s in cell parameters are only used when they are defined by crystal symmetry. An approximate (isotropic) treatment of cell e.s.d.'s is used for estimating e.s.d.'s involving l.s. planes.

### Fractional atomic coordinates and isotropic or equivalent isotropic displacement parameters (Å^2^)

**Table:**

	*x*	*y*	*z*	*U*_iso_*/*U*_eq_	
O1	0.83856 (13)	0.82806 (10)	0.70740 (9)	0.0232 (3)	
O2	0.77000 (13)	0.93616 (10)	0.53428 (9)	0.0240 (3)	
O3	1.55559 (14)	0.19841 (12)	0.52637 (11)	0.0352 (3)	
O4	1.53950 (14)	0.26192 (12)	0.36149 (10)	0.0320 (3)	
O5	0.70198 (12)	0.97785 (10)	0.86081 (9)	0.0229 (3)	
O6	0.86075 (12)	0.93425 (10)	1.03307 (9)	0.0217 (3)	
O7	0.08469 (14)	1.67994 (12)	1.06765 (12)	0.0343 (3)	
O8	0.21823 (14)	1.70388 (11)	1.22686 (10)	0.0331 (3)	
N1	1.05331 (17)	0.49336 (14)	0.73956 (12)	0.0312 (4)	
N2	1.51106 (15)	0.25875 (13)	0.45509 (12)	0.0241 (3)	
N3	0.34748 (15)	1.21226 (13)	0.81431 (11)	0.0231 (3)	
N4	0.17862 (16)	1.65717 (13)	1.13420 (12)	0.0250 (3)	
C1	1.04870 (18)	0.72332 (15)	0.52312 (13)	0.0206 (4)	
C2	0.99041 (17)	0.73654 (14)	0.61828 (13)	0.0199 (4)	
H2	1.0132	0.6944	0.6813	0.024*	
C3	0.90092 (17)	0.80994 (14)	0.62037 (12)	0.0186 (4)	
C4	0.86490 (17)	0.87172 (14)	0.52605 (13)	0.0193 (4)	
C5	0.92618 (18)	0.86239 (15)	0.43441 (13)	0.0207 (4)	
H5	0.9058	0.9065	0.3723	0.025*	
C6	1.01697 (18)	0.78904 (15)	0.43308 (13)	0.0214 (4)	
H6	1.0583	0.7834	0.3698	0.026*	
C7	0.86525 (19)	0.76349 (16)	0.80335 (13)	0.0243 (4)	
H7A	0.8146	0.7843	0.8594	0.036*	
H7B	0.9620	0.7800	0.8334	0.036*	
H7C	0.8374	0.6820	0.7828	0.036*	
C8	0.72379 (19)	0.99399 (16)	0.43758 (13)	0.0259 (4)	
H8A	0.6562	1.0379	0.4537	0.039*	
H8B	0.6834	0.9379	0.3766	0.039*	
H8C	0.8003	1.0455	0.4165	0.039*	
C9	1.13862 (18)	0.64400 (15)	0.51114 (13)	0.0222 (4)	
H9	1.1877	0.6586	0.4521	0.027*	
C10	1.16493 (18)	0.55272 (15)	0.56936 (13)	0.0211 (4)	
C11	1.10295 (18)	0.52212 (15)	0.66500 (13)	0.0215 (4)	
C12	1.25355 (17)	0.47620 (14)	0.53921 (13)	0.0188 (4)	
C13	1.29928 (17)	0.39916 (14)	0.61403 (13)	0.0198 (4)	
H13	1.2727	0.3960	0.6846	0.024*	
C14	1.38285 (17)	0.32729 (15)	0.58691 (13)	0.0204 (4)	
H14	1.4135	0.2749	0.6380	0.024*	
C15	1.42069 (17)	0.33351 (14)	0.48391 (13)	0.0199 (4)	
C16	1.37606 (17)	0.40682 (15)	0.40728 (13)	0.0214 (4)	
H16	1.4025	0.4085	0.3366	0.026*	
C17	1.29240 (17)	0.47784 (15)	0.43440 (13)	0.0205 (4)	
H17	1.2606	0.5284	0.3818	0.025*	
C20	0.61948 (17)	1.17523 (14)	1.06498 (13)	0.0185 (3)	
C21	0.61639 (17)	1.11728 (15)	0.96284 (13)	0.0198 (4)	
H21	0.5576	1.1331	0.8989	0.024*	
C22	0.69803 (17)	1.03785 (14)	0.95513 (12)	0.0187 (4)	
C23	0.78556 (17)	1.01356 (14)	1.04955 (13)	0.0186 (4)	
C24	0.78929 (17)	1.07024 (15)	1.15006 (13)	0.0205 (4)	
H24	0.8479	1.0541	1.2140	0.025*	
C25	0.70765 (17)	1.15032 (15)	1.15751 (13)	0.0204 (4)	
H25	0.7116	1.1890	1.2267	0.024*	
C26	0.62349 (19)	1.00501 (16)	0.76028 (13)	0.0241 (4)	
H26A	0.6351	0.9559	0.6990	0.036*	
H26B	0.6537	1.0849	0.7454	0.036*	
H26C	0.5281	0.9929	0.7676	0.036*	
C27	0.95890 (18)	0.91253 (16)	1.12473 (13)	0.0236 (4)	
H27A	1.0062	0.8538	1.1021	0.035*	
H27B	0.9133	0.8863	1.1863	0.035*	
H27C	1.0241	0.9827	1.1483	0.035*	
C28	0.53948 (17)	1.26103 (15)	1.08144 (13)	0.0200 (4)	
H28	0.5597	1.2965	1.1537	0.024*	
C29	0.44121 (17)	1.29992 (14)	1.01262 (12)	0.0179 (3)	
C30	0.39106 (17)	1.24995 (14)	0.90229 (13)	0.0179 (3)	
C31	0.37513 (17)	1.39410 (14)	1.04263 (12)	0.0177 (3)	
C32	0.25478 (18)	1.41207 (15)	0.97922 (13)	0.0209 (4)	
H32	0.2156	1.3634	0.9158	0.025*	
C33	0.19096 (18)	1.49957 (15)	1.00682 (14)	0.0229 (4)	
H33	0.1105	1.5126	0.9619	0.028*	
C34	0.24767 (17)	1.56705 (14)	1.10128 (13)	0.0204 (4)	
C35	0.36790 (18)	1.55373 (14)	1.16528 (13)	0.0199 (4)	
H35	0.4058	1.6023	1.2290	0.024*	
C36	0.43196 (18)	1.46850 (14)	1.13491 (13)	0.0194 (4)	
H36	0.5160	1.4600	1.1772	0.023*	

### Atomic displacement parameters (Å^2^)

**Table:**

	*U*^11^	*U*^22^	*U*^33^	*U*^12^	*U*^13^	*U*^23^
O1	0.0321 (7)	0.0269 (7)	0.0152 (5)	0.0114 (6)	0.0105 (5)	0.0017 (5)
O2	0.0294 (7)	0.0285 (7)	0.0184 (6)	0.0152 (6)	0.0064 (5)	0.0021 (5)
O3	0.0390 (9)	0.0396 (8)	0.0343 (7)	0.0228 (7)	0.0102 (6)	0.0074 (6)
O4	0.0344 (8)	0.0400 (8)	0.0257 (6)	0.0113 (6)	0.0132 (6)	−0.0049 (6)
O5	0.0274 (7)	0.0286 (7)	0.0150 (5)	0.0121 (6)	0.0034 (5)	−0.0039 (5)
O6	0.0244 (7)	0.0258 (7)	0.0184 (6)	0.0130 (5)	0.0052 (5)	0.0013 (5)
O7	0.0304 (8)	0.0300 (8)	0.0450 (8)	0.0147 (6)	0.0043 (6)	−0.0018 (6)
O8	0.0397 (8)	0.0300 (8)	0.0324 (7)	0.0083 (6)	0.0131 (6)	−0.0085 (6)
N1	0.0406 (10)	0.0357 (10)	0.0234 (7)	0.0156 (8)	0.0132 (7)	0.0058 (7)
N2	0.0222 (8)	0.0264 (8)	0.0246 (7)	0.0052 (7)	0.0062 (6)	−0.0032 (6)
N3	0.0266 (8)	0.0261 (8)	0.0179 (7)	0.0080 (7)	0.0043 (6)	0.0004 (6)
N4	0.0249 (8)	0.0212 (8)	0.0317 (8)	0.0057 (7)	0.0120 (7)	−0.0003 (6)
C1	0.0233 (9)	0.0210 (9)	0.0181 (8)	0.0041 (7)	0.0056 (7)	−0.0005 (6)
C2	0.0232 (9)	0.0209 (9)	0.0161 (7)	0.0041 (7)	0.0045 (7)	0.0015 (6)
C3	0.0220 (9)	0.0196 (9)	0.0145 (7)	0.0016 (7)	0.0064 (6)	−0.0025 (6)
C4	0.0210 (9)	0.0189 (9)	0.0181 (8)	0.0043 (7)	0.0038 (6)	−0.0025 (6)
C5	0.0255 (10)	0.0225 (9)	0.0148 (7)	0.0059 (7)	0.0036 (7)	0.0022 (6)
C6	0.0246 (9)	0.0252 (9)	0.0163 (7)	0.0052 (7)	0.0079 (7)	0.0002 (6)
C7	0.0322 (10)	0.0275 (10)	0.0154 (7)	0.0067 (8)	0.0092 (7)	0.0018 (7)
C8	0.0324 (11)	0.0286 (10)	0.0197 (8)	0.0152 (8)	0.0027 (7)	0.0025 (7)
C9	0.0206 (9)	0.0270 (10)	0.0191 (8)	0.0023 (7)	0.0057 (7)	−0.0010 (7)
C10	0.0214 (9)	0.0264 (9)	0.0167 (8)	0.0063 (7)	0.0047 (7)	−0.0001 (7)
C11	0.0247 (10)	0.0245 (9)	0.0173 (8)	0.0100 (8)	0.0034 (7)	−0.0001 (7)
C12	0.0186 (9)	0.0209 (9)	0.0163 (7)	0.0020 (7)	0.0037 (6)	−0.0009 (6)
C13	0.0220 (9)	0.0231 (9)	0.0144 (7)	0.0032 (7)	0.0047 (6)	−0.0002 (6)
C14	0.0211 (9)	0.0208 (9)	0.0188 (8)	0.0032 (7)	0.0030 (7)	0.0013 (6)
C15	0.0172 (9)	0.0214 (9)	0.0209 (8)	0.0032 (7)	0.0038 (6)	−0.0048 (7)
C16	0.0206 (9)	0.0285 (10)	0.0151 (7)	0.0027 (7)	0.0054 (6)	−0.0010 (7)
C17	0.0239 (9)	0.0236 (9)	0.0144 (7)	0.0037 (7)	0.0045 (7)	0.0017 (6)
C20	0.0190 (9)	0.0212 (9)	0.0173 (7)	0.0060 (7)	0.0061 (6)	0.0022 (6)
C21	0.0206 (9)	0.0242 (9)	0.0155 (7)	0.0060 (7)	0.0038 (6)	0.0010 (6)
C22	0.0210 (9)	0.0214 (9)	0.0147 (7)	0.0034 (7)	0.0063 (6)	−0.0006 (6)
C23	0.0196 (9)	0.0196 (9)	0.0184 (8)	0.0053 (7)	0.0068 (7)	0.0020 (6)
C24	0.0200 (9)	0.0259 (9)	0.0172 (8)	0.0071 (7)	0.0044 (6)	0.0030 (7)
C25	0.0248 (9)	0.0235 (9)	0.0143 (7)	0.0065 (7)	0.0055 (7)	−0.0012 (6)
C26	0.0296 (10)	0.0298 (10)	0.0144 (7)	0.0101 (8)	0.0030 (7)	−0.0016 (7)
C27	0.0229 (10)	0.0286 (10)	0.0219 (8)	0.0119 (8)	0.0036 (7)	0.0027 (7)
C28	0.0241 (9)	0.0231 (9)	0.0148 (7)	0.0063 (7)	0.0069 (7)	0.0003 (6)
C29	0.0208 (9)	0.0210 (9)	0.0140 (7)	0.0054 (7)	0.0075 (6)	0.0018 (6)
C30	0.0203 (9)	0.0185 (9)	0.0179 (8)	0.0075 (7)	0.0071 (7)	0.0041 (6)
C31	0.0200 (9)	0.0210 (9)	0.0141 (7)	0.0048 (7)	0.0073 (6)	0.0047 (6)
C32	0.0239 (9)	0.0243 (9)	0.0157 (7)	0.0075 (7)	0.0036 (7)	−0.0007 (6)
C33	0.0221 (9)	0.0278 (10)	0.0211 (8)	0.0089 (8)	0.0051 (7)	0.0037 (7)
C34	0.0226 (9)	0.0185 (9)	0.0237 (8)	0.0064 (7)	0.0115 (7)	0.0025 (7)
C35	0.0244 (9)	0.0202 (9)	0.0163 (7)	0.0031 (7)	0.0079 (7)	0.0013 (6)
C36	0.0203 (9)	0.0234 (9)	0.0158 (7)	0.0053 (7)	0.0048 (6)	0.0031 (6)

### Geometric parameters (Å, °)

**Table:**

O1—C3	1.3639 (18)	C13—C14	1.385 (2)
O1—C7	1.435 (2)	C13—H13	0.9500
O2—C4	1.355 (2)	C14—C15	1.382 (2)
O2—C8	1.439 (2)	C14—H14	0.9500
O3—N2	1.2248 (19)	C15—C16	1.375 (2)
O4—N2	1.2308 (18)	C16—C17	1.379 (2)
O5—C22	1.3602 (18)	C16—H16	0.9500
O5—C26	1.432 (2)	C17—H17	0.9500
O6—C23	1.3528 (19)	C20—C25	1.400 (2)
O6—C27	1.438 (2)	C20—C21	1.414 (2)
O7—N4	1.226 (2)	C20—C28	1.450 (2)
O8—N4	1.2299 (19)	C21—C22	1.381 (2)
N1—C11	1.148 (2)	C21—H21	0.9500
N2—C15	1.471 (2)	C22—C23	1.410 (2)
N3—C30	1.148 (2)	C23—C24	1.388 (2)
N4—C34	1.472 (2)	C24—C25	1.387 (2)
C1—C6	1.396 (2)	C24—H24	0.9500
C1—C2	1.415 (2)	C25—H25	0.9500
C1—C9	1.453 (2)	C26—H26A	0.9800
C2—C3	1.377 (2)	C26—H26B	0.9800
C2—H2	0.9500	C26—H26C	0.9800
C3—C4	1.417 (2)	C27—H27A	0.9800
C4—C5	1.387 (2)	C27—H27B	0.9800
C5—C6	1.386 (2)	C27—H27C	0.9800
C5—H5	0.9500	C28—C29	1.355 (2)
C6—H6	0.9500	C28—H28	0.9500
C7—H7A	0.9800	C29—C30	1.444 (2)
C7—H7B	0.9800	C29—C31	1.481 (2)
C7—H7C	0.9800	C31—C32	1.395 (2)
C8—H8A	0.9800	C31—C36	1.404 (2)
C8—H8B	0.9800	C32—C33	1.390 (2)
C8—H8C	0.9800	C32—H32	0.9500
C9—C10	1.357 (2)	C33—C34	1.381 (2)
C9—H9	0.9500	C33—H33	0.9500
C10—C11	1.447 (2)	C34—C35	1.381 (2)
C10—C12	1.478 (2)	C35—C36	1.381 (2)
C12—C13	1.397 (2)	C35—H35	0.9500
C12—C17	1.408 (2)	C36—H36	0.9500
			
C3—O1—C7	117.62 (13)	C15—C16—H16	120.5
C4—O2—C8	117.01 (12)	C17—C16—H16	120.5
C22—O5—C26	117.68 (13)	C16—C17—C12	120.72 (16)
C23—O6—C27	117.60 (12)	C16—C17—H17	119.6
O3—N2—O4	123.80 (15)	C12—C17—H17	119.6
O3—N2—C15	118.02 (13)	C25—C20—C21	118.28 (15)
O4—N2—C15	118.16 (15)	C25—C20—C28	116.82 (14)
O7—N4—O8	124.42 (15)	C21—C20—C28	124.90 (15)
O7—N4—C34	117.73 (14)	C22—C21—C20	120.62 (15)
O8—N4—C34	117.85 (15)	C22—C21—H21	119.7
C6—C1—C2	118.54 (15)	C20—C21—H21	119.7
C6—C1—C9	116.93 (14)	O5—C22—C21	124.87 (15)
C2—C1—C9	124.53 (15)	O5—C22—C23	114.96 (14)
C3—C2—C1	120.58 (15)	C21—C22—C23	120.17 (14)
C3—C2—H2	119.7	O6—C23—C24	125.05 (15)
C1—C2—H2	119.7	O6—C23—C22	115.42 (13)
O1—C3—C2	125.12 (15)	C24—C23—C22	119.53 (15)
O1—C3—C4	114.88 (14)	C25—C24—C23	120.21 (15)
C2—C3—C4	119.99 (14)	C25—C24—H24	119.9
O2—C4—C5	125.28 (15)	C23—C24—H24	119.9
O2—C4—C3	115.28 (13)	C24—C25—C20	121.20 (14)
C5—C4—C3	119.44 (15)	C24—C25—H25	119.4
C6—C5—C4	120.24 (15)	C20—C25—H25	119.4
C6—C5—H5	119.9	O5—C26—H26A	109.5
C4—C5—H5	119.9	O5—C26—H26B	109.5
C5—C6—C1	121.08 (14)	H26A—C26—H26B	109.5
C5—C6—H6	119.5	O5—C26—H26C	109.5
C1—C6—H6	119.5	H26A—C26—H26C	109.5
O1—C7—H7A	109.5	H26B—C26—H26C	109.5
O1—C7—H7B	109.5	O6—C27—H27A	109.5
H7A—C7—H7B	109.5	O6—C27—H27B	109.5
O1—C7—H7C	109.5	H27A—C27—H27B	109.5
H7A—C7—H7C	109.5	O6—C27—H27C	109.5
H7B—C7—H7C	109.5	H27A—C27—H27C	109.5
O2—C8—H8A	109.5	H27B—C27—H27C	109.5
O2—C8—H8B	109.5	C29—C28—C20	131.78 (15)
H8A—C8—H8B	109.5	C29—C28—H28	114.1
O2—C8—H8C	109.5	C20—C28—H28	114.1
H8A—C8—H8C	109.5	C28—C29—C30	121.52 (15)
H8B—C8—H8C	109.5	C28—C29—C31	124.14 (14)
C10—C9—C1	131.43 (15)	C30—C29—C31	114.33 (14)
C10—C9—H9	114.3	N3—C30—C29	177.59 (18)
C1—C9—H9	114.3	C32—C31—C36	118.01 (15)
C9—C10—C11	121.11 (15)	C32—C31—C29	120.82 (15)
C9—C10—C12	123.52 (15)	C36—C31—C29	121.17 (15)
C11—C10—C12	115.34 (15)	C33—C32—C31	121.49 (16)
N1—C11—C10	177.26 (19)	C33—C32—H32	119.3
C13—C12—C17	118.40 (15)	C31—C32—H32	119.3
C13—C12—C10	120.50 (14)	C34—C33—C32	118.21 (16)
C17—C12—C10	121.09 (15)	C34—C33—H33	120.9
C14—C13—C12	121.04 (14)	C32—C33—H33	120.9
C14—C13—H13	119.5	C35—C34—C33	122.21 (15)
C12—C13—H13	119.5	C35—C34—N4	118.82 (15)
C15—C14—C13	118.55 (16)	C33—C34—N4	118.97 (16)
C15—C14—H14	120.7	C34—C35—C36	118.78 (15)
C13—C14—H14	120.7	C34—C35—H35	120.6
C16—C15—C14	122.18 (16)	C36—C35—H35	120.6
C16—C15—N2	118.92 (14)	C35—C36—C31	121.19 (16)
C14—C15—N2	118.90 (15)	C35—C36—H36	119.4
C15—C16—C17	119.09 (14)	C31—C36—H36	119.4
			
C6—C1—C2—C3	2.1 (3)	C25—C20—C21—C22	0.3 (3)
C9—C1—C2—C3	−176.79 (16)	C28—C20—C21—C22	179.07 (16)
C7—O1—C3—C2	−1.8 (2)	C26—O5—C22—C21	5.0 (2)
C7—O1—C3—C4	176.78 (15)	C26—O5—C22—C23	−175.15 (15)
C1—C2—C3—O1	179.45 (16)	C20—C21—C22—O5	179.90 (16)
C1—C2—C3—C4	1.0 (3)	C20—C21—C22—C23	0.1 (3)
C8—O2—C4—C5	3.9 (2)	C27—O6—C23—C24	−4.6 (2)
C8—O2—C4—C3	−175.80 (15)	C27—O6—C23—C22	175.45 (15)
O1—C3—C4—O2	−2.5 (2)	O5—C22—C23—O6	0.0 (2)
C2—C3—C4—O2	176.11 (15)	C21—C22—C23—O6	179.80 (15)
O1—C3—C4—C5	177.76 (15)	O5—C22—C23—C24	179.98 (15)
C2—C3—C4—C5	−3.6 (3)	C21—C22—C23—C24	−0.2 (3)
O2—C4—C5—C6	−176.56 (16)	O6—C23—C24—C25	179.92 (16)
C3—C4—C5—C6	3.1 (3)	C22—C23—C24—C25	−0.1 (3)
C4—C5—C6—C1	0.0 (3)	C23—C24—C25—C20	0.5 (3)
C2—C1—C6—C5	−2.6 (3)	C21—C20—C25—C24	−0.6 (3)
C9—C1—C6—C5	176.37 (16)	C28—C20—C25—C24	−179.45 (16)
C6—C1—C9—C10	−163.11 (19)	C25—C20—C28—C29	−175.78 (18)
C2—C1—C9—C10	15.8 (3)	C21—C20—C28—C29	5.4 (3)
C1—C9—C10—C11	−2.8 (3)	C20—C28—C29—C30	4.5 (3)
C1—C9—C10—C12	175.28 (17)	C20—C28—C29—C31	−176.36 (17)
C9—C10—C12—C13	166.39 (17)	C28—C29—C31—C32	−164.68 (16)
C11—C10—C12—C13	−15.4 (2)	C30—C29—C31—C32	14.5 (2)
C9—C10—C12—C17	−14.5 (3)	C28—C29—C31—C36	15.9 (3)
C11—C10—C12—C17	163.69 (16)	C30—C29—C31—C36	−164.91 (15)
C17—C12—C13—C14	1.1 (3)	C36—C31—C32—C33	−1.0 (2)
C10—C12—C13—C14	−179.74 (16)	C29—C31—C32—C33	179.49 (15)
C12—C13—C14—C15	0.2 (3)	C31—C32—C33—C34	−1.9 (3)
C13—C14—C15—C16	−1.3 (3)	C32—C33—C34—C35	3.2 (3)
C13—C14—C15—N2	179.07 (15)	C32—C33—C34—N4	−177.59 (15)
O3—N2—C15—C16	176.16 (16)	O7—N4—C34—C35	168.39 (16)
O4—N2—C15—C16	−2.5 (2)	O8—N4—C34—C35	−12.2 (2)
O3—N2—C15—C14	−4.2 (2)	O7—N4—C34—C33	−10.9 (2)
O4—N2—C15—C14	177.10 (16)	O8—N4—C34—C33	168.55 (16)
C14—C15—C16—C17	1.0 (3)	C33—C34—C35—C36	−1.4 (3)
N2—C15—C16—C17	−179.36 (15)	N4—C34—C35—C36	179.41 (15)
C15—C16—C17—C12	0.4 (3)	C34—C35—C36—C31	−1.8 (2)
C13—C12—C17—C16	−1.4 (3)	C32—C31—C36—C35	2.9 (2)
C10—C12—C17—C16	179.45 (16)	C29—C31—C36—C35	−177.60 (15)
